# A comparative study of the efficacy and safety of high-flow nasal cannula and nasal continuous positive airway pressure in neonatal respiratory distress syndrome: A protocol for systematic review and meta-analysis

**DOI:** 10.1097/MD.0000000000029109

**Published:** 2022-04-15

**Authors:** Jiang-Jiang Wang, Lei Zhang, Na Cai

**Affiliations:** The First People's Hospital of Jiangxia District, Wuhan City (Union Jiangnan Hospital Huazhong University of Science and Technology), Wuhan, Hubei, China.

**Keywords:** continuous positive airway pressure, efficacy, high-flow nasal cannula, neonatal respiratory distress syndrome

## Abstract

**Background:**

When it comes to preterm newborns, respiratory distress syndrome (RDS) is the most frequent respiratory condition. Despite the fact that it is well acknowledged that preterm delivery plays a significant role, the causes of lung damage are still not completely understood. In newborns with extremely low birth weight and neonatal RDS, nasal continuous positive airway pressure has been suggested as the first respiratory assistance for spontaneous breathing. In the current research, we aim to carry out a meta-analysis to assess the effectiveness and safety of high-flow nasal cannula (HFNC) and non-invasive continuous positive airway pressure (nCPAP) in patients with neonatal respiratory distress syndrome (NRDS).

**Methods:**

We intend to search the following databases: PubMed, EMBASE, Cochrane Library, Wanfang database, China National Knowledge Infrastructure (CNKI), and Google Scholar, starting from their initial publication until February 2022, to identify randomized controlled trials comparing HFNC to nCPAP in patients with NRDS. The suitable papers will be chosen by 2 writers who will work independently of one another. Using the Cochrane updated technique for risk of bias, each included article will be subjected to an independent data extraction process by the 2 writers who will then independently evaluate the risk of bias. Consequently, a third author will be asked to address any discrepancies that may arise between the writers. It will be necessary to pool the data and do a meta-analysis with the help of the RevMan 5.3 software.

**Results:**

In this study, the effectiveness and safety of HFNC will be compared with those of nCPAP in patients with NRDS.

**Conclusion:**

If the results of this research are confirmed, they may serve as a summary of the most recent data for non-invasive respiratory assistance in NRDS.

**Ethics and dissemination:**

The study will require ethical approval.

**Registration number:**

DOI 10.17605/OSF.IO/BKSQ5

## 1 Introduction

Neonatal respiratory distress syndrome (RDS) is characterized by the presence of clinical indications of early neonatal respiratory distress, consistent radiologic characteristics, and the need for supplemental oxygen to maintain a saturation >85% during the first 24 hours after delivery.^[1,2]^ When compared with other illnesses, it is more probable to bring about morbidity and death in infants and children,^[3]^ and it continues to be a serious public health concern. Even those who survive RDS seem to be at greater risk of developing serious sequelae.^[2]^ Low gestational age, male gender, maternal age, maternal chorioamnionitis, and multifetal pregnancy are all actual or prospective risk factors for RDS.^[4,5]^

The introduction of surfactant has a significant influence on the outcomes of neonatal respiratory distress syndrome (NRDS).^[6]^ In the recent decade, there has been a significant change in the practise of surfactant therapy, with studies suggesting that early selected rescue treatment is superior to the previously performed prophylactic dosing when compared with prior practise.^[7]^ The utilization of oxygen therapy in the treatment of NRDS is quite beneficial. Non-invasive continuous positive airway pressure (nCPAP) is the most often utilized non-invasive breathing treatment for NRDS, and it has been shown to be effective in treating the condition.^[8,9]^ The drug's tolerance and compliance are low, however, since it is easily associated with newborn nasal damage and screaming. An innovative non-invasive respiratory assistance technique, the high-flow nasal cannula (HFNC), is being developed. In order to cleanse the anatomically inefficient cavity of the nasopharynx, it is necessary to administer warm and humidified air–oxygen combination.^[10]^

This will enhance respiratory tract conductivity, lower upper respiratory tract inspiratory resistance, and increase lung ventilation rate. When HFNC and nCPAP were evaluated as respiratory support strategies for NRDS, several systematic assessments revealed that both approaches produced the same therapeutic impact and were effective respiratory support methods.^[11,12]^ Although some researchers feel that HFNC might delay the time required for endotracheal intubation, it has also been linked to an increase in the frequency of complications and a higher fatality rate in patients.^[13,14]^ There is no agreement on whether HFNC should be the primary treatment for NRDS.^[15]^ Because of the inconsistency of the above research findings, the present study will be conducted in order to further systematically evaluate the efficacy and safety of HFNC compared with nCPAP in NRDS. The results of this study will be used to help clinicians use it more effectively in clinical practise.

## 2 Methods

This review has been registered on the Open Science Framework database with DOI number: 10.17605/OSF.IO/BKSQ5. The protocol follows the Preferred Reporting Items for Systematic Reviews and Meta-Analyses Protocols (PRISMA-P) statement guidelines.

## 3 Criteria for considering studies for this review

### 3.1 Types of studies

We will include any randomized controlled trials (RCTs) of HFNC against nCPAP in NRDS that were conducted in a language other than Chinese or English, regardless of whether blinding or allocation concealment was used. Non-randomized controlled trials, series of case reports, and cross-research will be eliminated from consideration.

### 3.2 Types of participants

Children born to mothers who were <37 weeks pregnant and who were diagnosed with RDS as a main diagnosis.

### 3.3 Types of interventions

The noninvasive breathing assistance was initiated during the first 24 hours of life for newborns with neurodevelopmental disorders. The study comprised neonates who had received surfactant in accordance with usual practise prior to the randomization. All randomized controlled trials that assessed any of the 2 noninvasive respiratory support techniques separately were disqualified from consideration.

### 3.4 Types of outcome measures

The key outcomes were as follows: the need for invasive mechanical ventilation during the first 7 days after randomization; and the failure of the therapy. The incidence of death, the incidence of bronchopulmonary dysplasia, the incidence of mortality or bronchopulmonary dysplasia, and the incidence of air leakage were all measured as secondary outcomes.

## 4 Search methods for the identification of studies

### 4.1 Electronic searches

The following databases will be searched: PubMed, EMBASE, Cochrane Library, Wanfang database, China National Knowledge Infrastructure, and Google Scholar, starting from their inceptions until February 2022, to identify RCTs comparing HFNC to nCPAP in patients with NRDS. We will use the following search phrases, either alone or in combination, to find relevant articles: “neonatal respiratory distress syndrome,” “continuous positive airway pressure,” “high-flow nasal cannula,” and “randomised controlled trial.”

### 4.2 Searching other resources

Electronic searches will be conducted for relevant meta-analyses of RCTs comparing the efficacy and safety of HFNC compared with nCPAP in NRDS. Furthermore, we will filter relevant medical publications and periodicals in order to uncover material that is not already included in electronic database search results.

## 5 Data collection and analysis

### 5.1 Selection of studies

The titles and abstracts of all papers identified by the combined database searches will be integrated using Endnote X9, a reference management software programme, and any duplicates will be deleted from the merged database search results. One author will independently check search results against eligibility criteria in order to ensure that they meet the requirements. All studies that match the eligibility requirements based on the screening titles and abstracts will be sourced and read in full, unless they meet the criteria for exclusion. As part of the validity-enhancing process, another author will analyze and screen the titles and findings to ensure that they meet all qualifying requirements. A third author will be consulted in order to address any discrepancies that may arise between the writers.

### 5.2 Data extraction

The following information will be retrieved by 2 writers separately, using a data collecting table that has been previously prepared. In this study, information regarding study details, participant details, research techniques used, intervention strategies employed in both the treatment and control groups, as well as main and secondary results will be extracted. Whenever we discover that the data shown above are incomplete, we will contact the associated authors for more information. A third author will be consulted in order to address any discrepancies that may arise between the writers.

### 5.3 Assessment of risk of bias in included studies

Two authors will independently analyze each included research, using the domain-based evaluation method outlined by the Cochrane Handbook for Systematic Reviews of Interventions as the guideline for conducting systematic reviews. Their findings will be evaluated in the following areas: randomization sequence creation, allocation concealment, blinding of outcome assessors, inadequate outcome data, selective result reporting, and other sources of bias. Depending on its importance, each domain will be split into 3 categories: low, unclear, and high. A third author will be consulted in order to address any discrepancies that may arise between the authors.

### 5.4 Measures of treatment effect

We intend to record the mean difference or the standardized mean difference as well as the 95% confidence interval for each participant for continuous variable outcomes. In contrast, for dichotomous outcomes, we will record the relative risk and the 95% confidence interval.

### 5.5 Assessment of heterogeneity

The Chi-square test and *I*^2^ values will be used to determine whether or not there is heterogeneity. It will be regarded tolerable heterogeneity if *I*^2^ is <50%, and the pooled data will be pooled by applying a fixed-effect model. If *I*^2^ is >50%, considerable heterogeneity will be evaluated, and data will be pooled using a random-effects model to account for it.

### 5.6 Assessment of publication bias

If there are >10 randomized controlled trials included, a funnel plot will be used to identify any potential publishing biases, and an Egg regression test will be used to determine whether the funnel plot is symmetric.

## 6 Discussion

To the best of our knowledge, this systematic review is the first to investigate the effectiveness and safety of HFNC in NRDS when compared with nCPAP. It will include a complete overview of the existing information related to non-invasive respiratory support in NRDS, increasing functional status, quality of life, as well as the psychosocial effects of NRDS, and will be updated when new data becomes available. It is possible that this research will be useful to clinical practise, patients, and health policy makers in determining the effectiveness and safety of non-invasive respiratory assistance in patients with NRDS.

## Author contributions

**Conceptualization:** Jiang-Jiang Wang, Na Cai.

**Data curation:** Jiang-Jiang Wang, Lei Zhang, Na Cai.

**Formal analysis:** Jiang-Jiang Wang.

**Funding acquisition:** Lei Zhang, Na Cai.

**Investigation:** Jiang-Jiang Wang, Lei Zhang.

**Methodology:** Lei Zhang, Na Cai.

**Project administration:** Jiang-Jiang Wang, Lei Zhang.

**Resources:** Na Cai.

**Software:** Jiang-Jiang Wang, Lei Zhang.

**Supervision:** Jiang-Jiang Wang, Lei Zhang.

**Validation:** Jiang-Jiang Wang, Lei Zhang.

**Visualization:** Jiang-Jiang Wang, Lei Zhang, Na Cai.

**Writing – original draft:** Jiang-Jiang Wang.

**Writing – review & editing:** Lei Zhang, Na Cai.
